# Ontogenic Development of Digestive Enzymes in Mealworm Larvae (*Tenebrio molitor*) and Their Suitable Harvesting Time for Use as Fish Feed

**DOI:** 10.3390/insects11060393

**Published:** 2020-06-26

**Authors:** Somrak Rodjaroen, Karun Thongprajukaew, Puridet Khongmuang, Saowalak Malawa, Kimhun Tuntikawinwong, Suktianchai Saekhow

**Affiliations:** 1 Department of Agriculture, Faculty of Science and Technology, Nakhon Si Thammarat Rajabhat University, Nakhon Si Thammarat 80280, Thailand; somrak_rod@nstru.ac.th (S.R.); Poopooridet@gmail.com (P.K.); 2 Department of Applied Science, Faculty of Science, Prince of Songkla University, Songkhla 90112, Thailand; saowalak4866@gmail.com (S.M.); chaung_17@hotmail.com (S.S.); 3 Hatyaiwittayalai School, Songkhla 90110, Thailand; mingzapingin@gmail.com

**Keywords:** economic fish, harvesting time, in vitro digestibility, live feed, proximate composition

## Abstract

Mealworm larvae (*Tenebrio molitor*) are edible insects consumed in feed and food. In the current study, the optimal harvesting time of mealworm larvae for use as aquafeed was investigated during the ages of 30–90 days after hatching (DAH). Development of digestive enzymes, proximate composition, and in vitro protein digestibility using digestive enzymes from African catfish (*Clarias gariepinus*) and Nile tilapia (*Oreochromis niloticus*), were used as criteria. The specific activities of pepsin and trypsin significantly decreased with age (*p* < 0.05) from the first harvesting time until 50 and 45 DAH, respectively, while steadiness in these enzyme activities was observed onwards. Chymotrypsin specific activity appeared constant across all harvesting times. The specific activity of amylase significantly decreased in the later stages of development, while cellulase exhibited a different pattern suggesting it has a major role in dietary fiber utilization relative to starch. Regarding proximate compositions of the mealworm larvae, the moisture and ash contents decreased significantly with age, while the protein content exhibited the opposite trend with the highest contents from 60 to 90 DAH. Crude lipid was generally fairly constant, but its lowest value was observed in the earliest stage. In vitro protein digestibility was not significantly different across all harvesting times for both fish species, except for the significantly decreased digestibility value at 65 DAH relative to 30 and 35 DAH for Nile tilapia. However, based on the economic benefits of time for growth increment and proximate chemical composition, approximately 60 DAH is proposed as suitable for harvesting mealworm larvae to be used in fish feed.

## 1. Introduction

Mealworm larvae (*Tenebrio molitor*) are edible insects consumed by people in many Asian countries, particularly in Southeast Asia [1]. They are generally found as street food alongside other edible insects, and they can be applied in making a healthy snack food. Moreover, they are typically used as live feed for pets and zoo animals, including birds, reptiles, small mammals, amphibians, and fish, and they can be used in canned, dried or powdered forms [2,3]. For aquatic animals, mealworm larvae have been used to feed various species, such as African catfish, *Clarias gariepinus* [4]; Atlantic salmon, *Salmo salar* [5]; blackspot seabream, *Pagellus bogaraveo* [6]; gilthead sea bream, *Sparus aurata* [7]; Nile tilapia, *Oreochromis niloticus* [8]; and red sea bream, *Pagrus major* [9]. Therefore, mealworm larvae have received growing interest in the list of potential protein sources for food and feed.

The lifespan of mealworm beetles varies from 280 to 630 days, but the matured larval stage is typically reached 3–4 months after hatching at 18–20 °C [3]. These time profiles, as well as the nutritive values, also vary by the types of feed [3]. Therefore, juvenile hormones can be added to the food to prevent the molting of the larvae into adults, prolonging the harvesting time, as well as achieving higher weight and length of the larvae [10]. However, across a wide range of larval stages, nutritional information is unavailable for use as a criterion in selecting a suitable harvesting time range. Ontogenic development has been studied to understand the physiological changes and gut functions related to the growth and development of reared animal species. Across various studies, digestive enzymes are typically investigated since they are associated with nutritional status and feeding habits [11,12,13,14,15]. However, only limited information is available regarding the development of digestive enzymes in insects, in the literature reviewed.

In vitro digestibility (IVD) technique has been used to screen feedstuff or feed for animal rearing [15,16,17]. In terms of protein utilization, this technique detects the liberation of amino acids after proteolytic digestion. Therefore, this current study aimed to describe the ontogenic development of digestive enzymes in mealworm larvae, and we also used the IVD technique, proximate chemical composition, and growth performance as criteria for selecting a suitable harvesting time range for mealworm larvae. Findings from the current study provide basic knowledge of nutritional and physiological changes across a wide range of mealworm larval stages and can serve as practical guidelines for preparing mealworm larvae as fish feed. 

## 2. Materials and Methods

Preparation, rearing and harvesting of all animals in the current study conformed to the “Ethical Principles and Guidelines for the Use of Animals for Scientific Purposes”, National Research Council, Thailand (Application No. U1-06514-2560). Ten-day-old mealworm larvae were purchased from a private farm in the Nakhon Si Thammarat province of Thailand. They were reared in three plastic containers (30 cm width × 40 cm length × 10 cm height) containing 1 kg of wheat bran each. Two equal pieces of a ripe banana (*Musa acuminata* × *Musa balbisiana*) were placed on wheat bran to serve as a source of water and additives. The mealworm larvae were reared at a stocking density of 300 individuals per container, under a natural diurnal cycle (12-h dark/12-h light). Feces and dead mealworm larvae were removed from the container, and then new wheat bran was added to maintain 1 kg per container. The mealworm larvae were harvested at 30, 35, 40, 45, 50, 55, 60, 65, 70, 75, 80, 85 and 90 days after hatching (DAH). They were fasted for 12 h prior to sampling. All the samples were packed in polyethylene bags and chilled on ice for 30 min. At each harvesting time, twenty mealworm larvae were sampled while ten mealworm larvae (*n* = 10 per container) were weighed on a digital microbalance (Ohaus AR2140; Ohaus Corp., Parsippany, NJ, USA), and their lengths were measured to the nearest 0.01 mm using a digital Vernier caliper (Mitutoyo-500; Mitutoyo Corp., Kanagawa, Japan).

Undesirable contamination was removed carefully from the mealworm larvae, and then they were soaked in 5 mg L^−1^ of KMnO_4_ for 30 min [18,19], rinsed with distilled water three times, and filtered to remove the water. The whole bodies of the mealworm larvae were homogenized in cold 0.2 M phosphate buffer (pH 8, 1: 3 *w*/*v*) using a micro-homogenizer (THP-220; Omni International, Kennesaw, GA, USA). The homogenate was centrifuged at 15,000× *g* for 30 min at 4 °C, and then the supernatant was collected, aliquoted, and kept at −20 °C until use. The concentration of protein in the crude enzyme extract was assayed using the method of Lowry et al. [20], which used bovine serum albumin as standard protein. The concentration of soluble proteins (mg mL^−1^) was used to quantify the enzyme-specific activities (U mg protein^−1^). All assays were performed within one month after extraction.

Pepsin activity was determined based on the method of Worthington [21] using hemoglobin as the substrate. The assay conditions were set at pH 2 and 30 °C [22]. One unit (U) of pepsin activity was defined by an increase of 1.0 in absorbance at 280 nm. The trypsin and chymotrypsin activities were assayed according to Rungruangsak-Torrissen et al. [23], using *N*-benzoyl-*L*-Arg-*p*-nitroanilide and *N*-succinyl-Ala-Ala-Pro-Phe-*p*-nitroanilide as the substrates, respectively. The assay conditions were pH 8.5 and 55 °C [24], and pH 9.5 and 51 °C [12], for the respective enzymes. The linear response range to *p*-nitroanilide at 410 nm was used to quantify the activities of both enzymes. The amylase activity was determined based on the method of Bernfeld [25], using soluble starch as the substrate, in comparison to a maltose standard curve at 540 nm. The assay conditions were pH 5.4 and 25 °C [13]. Cellulase activity was determined according to the method of Mendels and Weber [26], using carboxymethylcellulose as substrate, in comparison to a glucose standard curve at 540 nm. The assay conditions were pH 8 and 30 °C [15]. One unit activity for all the digestive enzymes, except for pepsin, was defined as the amount that catalyzed the conversion of 1 μmol of substrate per minute.

Whole larval mealworm carcasses (*n* = 3 pooled sample per treatment) were minced and analyzed for moisture and ash contents according to standard methods of the AOAC [27]. Crude protein was determined by the method of Rungruangsak-Torrissen [28] after extracting it with TRIzol^TM^ reagent. Crude lipid was determined by extracting dried mealworm larval samples with ethyl acetate for 2 h in a rotary mixer, as described in Supannapong et al. [29]. All values were expressed on a wet weight basis. 

For the IVD test, thirty Nile tilapia (1.39 ± 0.28 g body weight) and African catfish (5.45 ± 0.24 g body weight) were purchased from a private farm in Songkhla province of Thailand. The fish were fed a commercial diet containing 18 and 25% crude protein (Charoen Pokphand Foods PCL., Bangkok, Thailand), respectively, and they were fasted for 12 h prior to sampling. The fish were sacrificed by chilling in ice, and only the intestine was carefully removed, weighed, and then used for digestive enzyme extraction. Three pooled intestinal samples (ten fish per sample) were extracted in 0.2 M Na_2_HPO_4_-NaH_2_PO_4_ buffer (pH 8) at a ratio of 1: 6 (*w*/*v*), using a micro-homogenizer (THP-220; Omni International, Kennesaw GA, USA). Centrifugation was performed at 15,000× *g* for 30 min at 4 °C prior to collecting the supernatant. The aliquots were kept at −20 °C until used for the IVD assay.

Fresh mealworm larvae were freeze-dried for 24 h using a freeze dryer (Flexidry; SP Scientific, Warminster, PA, USA) to eliminate moisture, and then they were minced to a homogeneous powder. The crude digestive enzymes from fish were dialyzed overnight against the extraction buffer. The in vitro protein digestibility procedure was performed as described by Thongprajukaew et al. [30]. The reaction mixtures contained 5 mg freeze-dried mealworm larvae powder, 50 μL of 0.5% chloramphenicol, and 125 μL crude enzyme extract from the fish. Ten milliliters of 50 mM Na_2_HPO_4_-NaH_2_PO_4_ buffer (pH 8.2) was added to perform the alkaline condition, contributing the near-optimal pH for digesting by fish trypsin. These mixtures were incubated at 30 °C under 200 rpm agitation for 24 h, against blank samples in which the enzyme volumes were replaced by extraction buffer. This condition provides a linear range of digested products (*DL*-alanine) after performing substrate: enzyme ratio (200: 1 *w*/*v*) for 24 h. The reaction was terminated by boiling at 100 °C for 10 min and then cooled down at room temperature for 30 min. The liberated amino acids in the digested solution were determined based on the trinitrobenzene sulfonic (TNBS) acid method, as described by Rungruangsak-Torrissen et al. [31]. The reaction contained 200 μL of the digested solution, 2 mL of 50 mM Na_2_HPO_4_-NaH_2_PO_4_ buffer (pH 8.2), and 1 mL of 0.1% TNBS. These mixtures were incubated in the dark at 60 °C for 1 h and then stopped by adding 1 mL of 1 M HCl. The colorimetric detection was measured spectrophotometrically at 420 nm against a linear range of standard *DL*-alanine. Trypsin activity in dialyzed crude enzyme extract from triplicate samples was analyzed [23] before standardizing the digestibility value (500 U for Nile tilapia and African catfish). The IVD of protein was expressed as μmol *DL*-alanine equivalent g mealworm larvae^−1^.

The experiment followed a completely randomized design (13 treatments × 3 replications). All the data were analyzed using the Statistical Package for Social Sciences, Version 14 (SPSS Inc., Chicago, IL, USA) and were reported as mean ± standard error of the mean (SEM). The normality and homogeneity of variance were checked. Means were compared using One-Way ANOVA, and differences were statistically analyzed with Duncan’s multiple range test and regarded as significant at *p* < 0.05 in all statistical analyses. Relationships between each pair of digestive enzymes were assessed from Pearson correlations.

## 3. Results

### 3.1. Changes in Weight and Length across Studied Period

**Figure 1:**
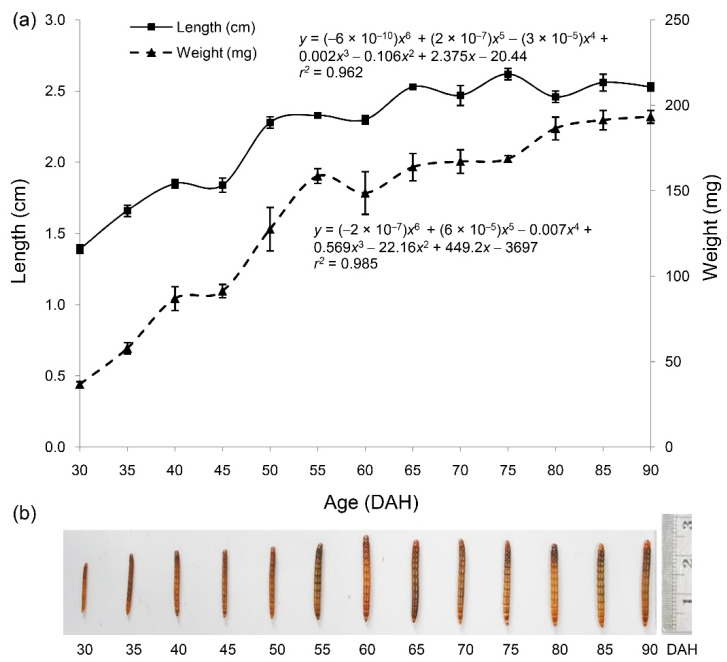
Ontogenic changes in body weight and length of mealworm larvae (**a**). Data are expressed as mean ± SEM from triplicate measurements (*n* = 30 per sampling time). Larval body lengths at different harvesting times (**b**). DAH stands for days after hatching.

The length and weight of the mealworm larvae increased progressively from 30 DAH on to 50 DAH and to 55 DAH, respectively (Figure 1a). A stable pattern of length was observed previously in mealworm larvae relative to their body weight. Both performance measures as functions of time (in DAH) were well fit by sixth-order polynomials, *y* = (−6 × 10^−10^)*x*^6^ + (2 × 10^−7^)*x*^5^ − (3 × 10^−5^)*x*^4^ + 0.002*x*^3^ − 0.106*x*^2^ + 2.375*x* − 20.44 and *y* = (−2 × 10^−7^)*x*^6^ + (6 × 10^−5^) − 0.007*x*^4^ + 0.569*x*^3^ − 22.16*x*^2^ + 449.2*x* − 3697, respectively. The larval body lengths and weights at 30 to 90 DAH are illustrated in Figure 1b.

### 3.2. Ontogenic Development of Digestive Enzymes

**Figure 2:**
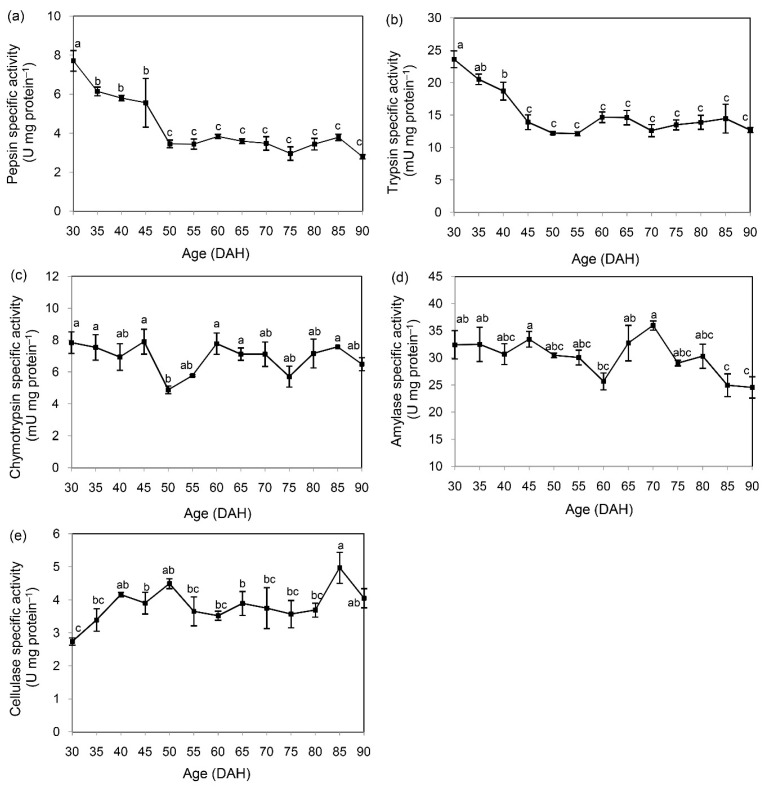
Ontogenic changes in specific activities of pepsin (**a**), trypsin (**b**), chymotrypsin (**c**), amylase (**d**), and cellulase (**e**) in mealworm larvae. Data are expressed as mean ± SEM from triplicate measurements. Significant differences between treatments are indicated by different superscripts (*p* < 0.05). DAH stands for days after hatching.

The specific activities of pepsin (Figure 2a) and trypsin (Figure 2b) significantly decreased with age (*p* < 0.05). High specific activity of both enzymes was observed from the first sampling time until 45 and 40 DAH, respectively, and then a steady level was maintained until 90 DAH. On the other hand, chymotrypsin specific activity appeared constant across all sampled times (Figure 2c). Relative to chymotrypsin, the specific activity of amylase exhibited a rather similar pattern, but with significantly decreased activity from 85 to 90 DAH (Figure 2d). For cellulase, the specific activity was lowest at 30 DAH, and then it was maintained at a steady level until the last sampling time (Figure 2e). Based on the Pearson correlation analysis, a significant relationship across all stages of mealworm larvae was observed between pepsin and trypsin and between pepsin and amylase, while in other pairs, the specific activities did not significantly correlate (Table 1).

### 3.3. Chemical Composition of Mealworm Larvae

Moisture and ash contents decreased significantly with the age of the mealworm larvae, while the protein content exhibited the opposite trend and remained high from 60 to 90 DAH. Crude lipid was generally similar across all stages of mealworm larvae, with the lowest value observed in the youngest stage (Table 2).

### 3.4. In Vitro Protein Digestibility Using Digestive Enzymes from Two Fish Species

**Figure 3:**
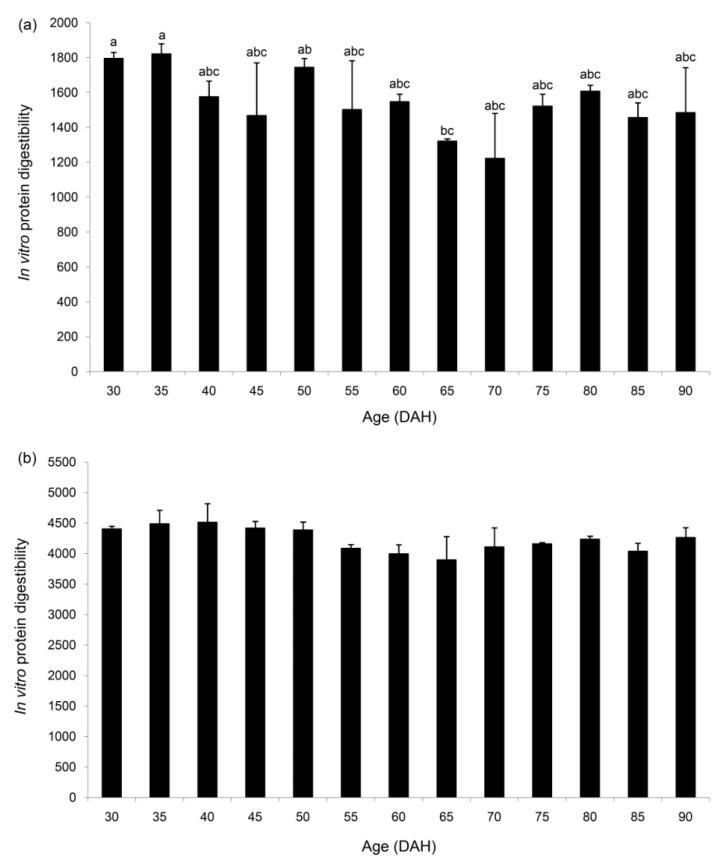
In vitro digestibility of protein (µmol *DL*-alanine equivalent g mealworm larvae^−1^) in mealworm larvae using digestive enzyme extract from Nile tilapia (**a**), and from African catfish (**b**). Data are expressed as mean ± SEM from triplicate measurements. Significant differences between treatments are indicated by different superscripts (*p* < 0.05). DAH stands for days after hatching.

In vitro digestibility (IVD) of protein, using digestive enzymes from Nile tilapia was generally similar across thirteen sampled times, but significantly decreased IVD was observed at 65 DAH relative to 30 and 35 DAH (Figure 3a). There were no effects of DAH on IVD for catfish (Figure 3b).

## 4. Discussion

The experiment was terminated at 90 DAH since some mealworm larvae were transitioning into the pupal stage. Weight and length of mealworm larvae fitted the polynomial regression models well, providing correlations of 0.962–0.985. These morphometric parameters were relatively constant from 65 and 80 DAH, respectively. These findings indicate that the growth rate was slower than linear, as the larvae spend their time eating and growing for the next transformation, concurrently with molting. This polynomial growth pattern has also been reported in the same species by Özsoy [32], as well as for various other insect species, such as melon thrips (*Thrips palmi*), soybean looper (*Pseudoplusia includens*), Mexican bean beetle (*Epilachna varivestis*), and velvetbean caterpillar (*Ancarsia gemmatalis*) [33,34].

Fly enzymes appear functionally to be similar to vertebrate enzymes [14]. In the current study, the ontogenic development has significant effects on specific activities of protein-digesting enzymes (pepsin-like, trypsin, and chymotrypsin) in mealworm larvae. Generally, pepsin and trypsin are major digestive enzymes in vertebrates, contributing to protein digestion in the stomach and intestine, respectively. However, the pepsin-like activity might occur due to substrate hydrolysis (hemoglobin) by other enzymes, such as cathepsin D, which is functionally equivalent and structurally similar to pepsin [35]. Specific inhibitor assays and the substrate-SDS PAGE procedure could be used to confirm the findings from this current study. Significant decreases in trypsin and pepsin-like activities occurred around 45 DAH and were maintained until the end of sampling, indicating maturation of gut functionality in terms of protein digestion, and also suggesting a shift in the feeding habits with maturation. In addition, the abrupt decreases in both enzyme activities with DAH suggest that protein intake plays a minor role in the older stages of mealworm larvae, so that feed sources low in protein might be appropriate. This hypothesis is also corroborated by the significant positive relationship (*r* = 0.761, *p* < 0.01, *n* = 39) between these enzymes throughout sampling. As regards chymotrypsin, this enzyme is activated by trypsin. Its relatively constant activity suggests a minor role of this enzyme in digesting protein, relative to pepsin-like enzymes or trypsin.

Physiological changes between protein and carbohydrate catabolism occur concurrently, as indicated by the significant positive relationship (*r* = 0.417, *p* < 0.01, *n* = 39) between the specific activities of pepsin and amylase. Over the sampled times, significant changes in carbohydrate-digesting enzymes (amylase and cellulase) were observed 10 days prior to the end of sampling, as compared to the youngest stage. These findings suggest that sources high in cellulosic materials might be more suitable than starch-based materials. This matches well with the proximate composition of wheat bran from the current study and the typical feed for this species (cereal bran, wheat flour, oat flour, or maize flour) supplemented with protein sources such as soybean flour, skimmed milk powder, or yeast [3,36].

The chemical composition of mealworm larvae is highly variable and influenced by the feed [3]. However, the composition of mealworm larvae based on the current study was similar and within the ranges in prior publications [1,10,37,38,39,40,41,42]. Fresh mealworm larvae contain approximately 60% moisture and high amounts of protein and lipid. Decreased moisture and ash contents with age might be due to the accumulation of protein, as well as the replacement of these constituents by chitin and chitosan, making mealworm larvae suitable as sources of oligosaccharides [43]. Not only a good amount of protein but also a good profile of amino acids has been reported [3]. Crude lipid was generally similar, but the lowest value was observed in the youngest stage. A low amount of lipid accumulation is reasonable for the youngest stage that initially exhibited the reserve. This finding is in agreement with the increased size of fat droplets within the trophocyte, as well as lipogenesis, following body weight increases [44]. However, the relatively high lipid content of dried mealworm larvae from the current study, as well as generally high level of unsaturated fatty acids, makes them susceptible to oxidation if not treated with an antioxidant [10,38].

IVD values depend on the quality of feedstuffs rather than their quantity [30,45,46]. IVD has been used to screen for suitable feedstuffs to feed fish. This technique is based on the digestion of protein by trypsin under alkaline conditions, mimicking the intestinal pH of fish [31,47]. The same technique from the current study has also been applied in feed development for various aquatic species, such as Atlantic salmon [28], Siamese fighting fish (*Betta splendens*) [30], Nile tilapia [46], silver barb (*Barbonymus gonionotus*) [46], and freshwater pearl mussel (*Hyriopsis* (*Hyriopsis*) *bialatus*) [29,48]. Based on our investigations, similar protein digestibility across all stages of mealworm larvae was observed, using digestive enzymes from Nile tilapia and African catfish. All the IVD values did not correlate with the proximate composition of protein in mealworm larvae. Comparing the IVD values was unsuitable for both fish species because of differences in nutrient digestibility, ages, genetics, feeding biology, and rearing conditions. In addition, limited information obtained from an assay performed in the current study may be caused by differences in digestive physiology between fish species. However, this finding supports the use of mealworm larvae as fish feed over a wide range of rearing (from 30 to 90 DAH), since they show a high apparent digestibility coefficient. This makes them a potential alternative feed for Nile tilapia fingerlings relative to other insect meals evaluated, such as speckled cockroach (*Nauphoeta cinerea*) meal, superworm (*Zophobas morio*) larvae meal, Madagascar hissing cockroach (*Gromphadorhina portentosa*) meal, and Jamaican field cricket (*Gryllus assimilis*) meal [49].

## 5. Conclusions

Digestive enzymes in mealworm larvae were genetically programed across thirteen developmental stages since only one type of feed was used throughout the experiment. Of the five digestive enzymes studied, carbohydrate-digesting enzymes (cellulase and amylase) play a major role in utilizing nutrients relative to protein-digesting enzymes (pepsin, trypsin, and chymotrypsin). The proximate composition of the mealworm larvae varied with the developmental stage, with protein being the major constituent, which is why they could be an excellent source of protein. However, mealworm larvae are not suitable as the main feed ingredient due to their high amounts of lipids, mainly unsaturated fatty acids, but they could be used in a mixture of different insects to obtain an ideal nutritional medium [1,42]. For use as fish feed, harvesting times had no or only minor effects on the IVD, based on our study. This suggests that a wide range of times is suitable for harvesting mealworm larvae (from 30 to 90 DAH). However, approximately 60 DAH is recommended due to the economic benefits of time for growth increment and proximate composition. Various factors can affect the validity or applicability of our findings including feed type and rearing conditions of the mealworm larvae. In addition, molting not only affects growth but also changes the components of the mealworm larvae before and after molting. Clarifying the relationships between molting and responsiveness of the digestive enzyme activities of the mealworm larvae, and/or the results of IVD should be of interest.

## Figures and Tables

**Table 1:** Pearson correlation coefficients (*r*) of time profiles of digestive enzyme specific activities, detected in mealworm larvae (*n* = 39).

Digestive Enzyme	Pepsin	Trypsin	Chymotrypsin	Amylase	Cellulase
Pepsin	1				
Trypsin	0.761 **	1			
Chymotrypsin	0.199	0.205	1		
Amylase	0.417 **	0.284	0.103	1	
Cellulase	–0.199	–0.159	0.244	0.187	1

Note: ** *p* < 0.01

**Table 2:** Proximate chemical compositions (in % of fresh weight) of mealworm larvae at each sampling time.

Age (DAH)	Moisture	Crude Protein	Crude Lipid	Ash
30	64.37 ± 0.83 ^a^	17.43 ± 3.34 ^e^	9.85 ± 0.17 ^e^	1.96 ± 0.06 ^a,b^
35	62.64 ± 1.24 ^a,b^	17.90 ± 1.82 ^d,e^	10.64 ± 1.21 ^d,e^	2.37 ± 0.55 ^a^
40	60.89 ± 0.24 ^b,c^	19.14 ± 0.65 ^d,e^	16.19 ± 0.98 ^a,b,c^	2.37 ± 0.28 ^a^
45	59.03 ± 0.17 ^c,d^	20.70 ± 1.04 ^c,d,e^	17.92 ± 2.53 ^a^	1.82 ± 0.08 ^b,c^
50	58.35 ± 0.46 ^d,e^	19.47 ± 0.61 ^d,e^	15.51 ± 1.43 ^a,b,c,d^	1.35 ± 0.11 ^c^
55	56.56 ± 0.44 ^e,f^	20.86 ± 0.92 ^c,d,e^	14.02 ± 0.70 ^a,b,c,d^	1.58 ± 0.08 ^b,c^
60	54.83 ± 0.42 ^f,g^	26.79 ± 0.45 ^a,b^	14.00 ± 2.18 ^a,b,c,d,e^	1.65 ± 0.04 ^b,c^
65	55.80 ± 1.38 ^f,g^	24.38 ± 1.91 ^a,b,c^	15.12 ± 0.75 ^a,b,c,d^	1.43 ± 0.09 ^c^
70	54.74 ± 1.41 ^f,g^	25.85 ± 2.25 ^a,b,c^	17.32 ± 0.50 ^a,b^	1.70 ± 0.10 ^b,c^
75	55.51 ± 0.79 ^f,g^	29.80 ± 1.05 ^a^	13.64 ± 2.07 ^a,b,c,d^	1.59 ± 0.05 ^b,c^
80	53.40 ± 0.43 ^g^	30.49 ± 2.76 ^a^	14.22 ± 0.20 ^a,b,c,d,e^	1.60 ± 0.11 ^b,c^
85	55.74 ± 0.32 ^f,g^	26.08 ± 1.45 ^a,b,c^	12.87 ± 0.16 ^b,c,d^	1.36 ± 0.14 ^c^
90	55.27 ± 0.69 ^f,g^	29.05 ± 0.97 ^a^	18.03 ± 2.28 ^a^	1.73 ± 0.06 ^b,c^
*p*-value	<0.001	<0.001	0.007	0.002

Note: DAH, days after hatching. Data are expressed as mean ± SEM (*n* = 3). Differences between means were tested with Duncan’s multiple range test. Different superscripts in the same column indicate a significant difference (*p* < 0.05).
